# Use of Point-of-care Ultrasound for Placement of a Gastric Tamponade Balloon

**DOI:** 10.5811/cpcem.24999

**Published:** 2025-02-15

**Authors:** Patrick Minges, Martina Diaz McDermott, Jazmyn Shaw

**Affiliations:** University of Cincinnati College of Medicine, Department of Emergency Medicine, Cincinnati, Ohio

**Keywords:** POCUS, gastric, varices, balloon tamponade device, hematemesis

## Abstract

**Case Presentation:**

A 30-year-old female with a history of alcoholic cirrhosis and esophageal varices presented with massive hematemesis. A gastric balloon tamponade device was subsequently placed to temporize variceal hemorrhage, and point-of-care ultrasound (POCUS) was used to confirm the appropriate placement of the gastric balloon before complete inflation. We describe a novel use of ultrasound for use in severely ill patients with gastrointestinal (GI) bleeding.

**Discussion:**

A fluid-filled and distended stomach has long been recognized as a cause of a false-positive focused assessment with sonography in trauma exam but may also be a vital piece of information in the scenario of a patient with suspected upper GI hemorrhage. There is very little description in the literature of using POCUS to confirm the appropriate placement of a gastric tamponade balloon with none by emergency physicians.. Ultrasound may offer advantages over plain radiography in this application given its speed and safety; thus, its utility for this task is worth further investigation.

## CASE PRESENTATION

A 30-year-old female presented to the emergency department (ED) with a chief complaint of hematemesis. She had a medical history of alcoholic cirrhosis complicated by ascites and esophageal varices. She had recently been admitted to another hospital and had banding of esophageal varices due to bleeding, requiring multiple blood transfusions. On arrival to the ED the patient was noted to be jaundiced and unwell appearing. Her initial vitals were blood pressure of 73/31 millimeters of mercury, heart rate 142 beats per minute, respiratory rate 32 breaths per minute, saturation 97% on room air, and she was afebrile. Her physical exam was notable for a distended, tender abdomen, and jaundice. While being examined, the patient was noted to have a large episode of coffee-ground emesis, approximately 800 milliliters (mL). Intravenous (IV) access was obtained, and one unit of packed red blood cells was infused. She was given octreotide, pantoprazole, and ceftriaxone.

**Image 1:**
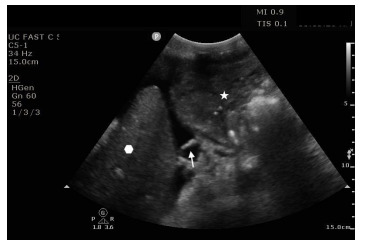
Subxiphoid/left upper quadrant image obtained with curvilinear 5-1 megahertz probe demonstrating the spleen (hexagon), the gastrosplenic ligament (arrow), and stomach with mixed echogenic contents (star) with anechoic free-fluid between the structures.

The patient had transient improvement in blood pressure, which dropped again as the initial unit of blood was completed. She had several further episodes of hematemesis. At this point, a focused assessment with sonography in trauma exam was performed due to patient instability and shock, which demonstrated anechoic free-fluid consistent with the patient’s known history of cirrhosis and varices. Also noted in the subxiphoid and left upper quadrant windows was a fluid-distended stomach with heterogeneous material concerning for blood products in the clinical scenario (Image 1).

At this time, large-bore central IV access was established, and massive transfusion protocol was started. The patient was intubated uneventfully for airway protection. The gastroenterology team was consulted, and a decision was made to place a Minnesota tube to temporize bleeding prior to transjugular intrahepatic portosystemic shunt (TIPS) procedure.

**Image 2:**
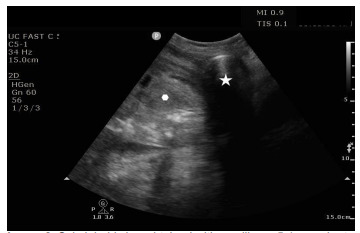
Subxiphoid view obtained with curvilinear 5-1 megahertz probe demonstrates upper portion of the stomach with mixed echogenic contents (hexagon) and rounded echogenic structure with posterior shadowing representing inflated gastric balloon of Minnesota tube (star).

After confirming the adequate function of Minnesota tube balloons and soaking it in an ice bath, a laryngoscope blade was placed in the oropharynx to visualize the endotracheal tube through the cords and obtain a view of the esophagus. The Minnesota tube was passed into the esophagus to a depth of 50 centimeters. At this point, the gastric balloon was inflated with 50 mL of air, and an ultrasound machine was used at the bedside to observe the stomach for the appearance of gaseous distension of the balloon (Image 2).

Once confirmed to be in the stomach, the gastric balloon was fully inflated and retracted until resistance was met, and gentle traction was applied by securing the tube with an endotracheal tube holder. After resuscitation, the patient was taken for emergent TIPS; unfortunately, she expired due to hemorrhagic shock.

**CPC-EM Capsule**

What do we already know about this clinical entity?

*The standard protocol for confirming correct placement of a gastric tamponade balloon is radiographic images*.

What is the major impact of the image(s)?

*A gastric tamponade balloon can be visualized in the stomach with ultrasound*.

How might this improve emergency medicine practice?

*Point-of-care ultrasound can be used to rapidly confirm safe placement of a gastric tamponade device in a clinically unstable patient*.

## DISCUSSION

Literature is sparse on the use of point-of-care ultrasound (POCUS) for guidance or confirmation of gastric tamponade device placement, with no reports of this application by emergency physicians.^1^,^2^ We describe a novel use of POCUS for this purpose. Although plain radiography is the preferred modality for confirmation of correct placement of a gastric balloon tamponade, POCUS offers significant potential advantages to plain radiography. It is more readily available, faster to interpret, and can be performed concurrently with other resuscitative procedures. Using POCUS for placement of gastric tubes is possible when other resources are unavailable, and research demonstrates reasonable accuracy for this purpose.^3^,^4^ Although use of POCUS for evaluation of balloon tamponade devices is not routine, it may be an alternative to plain radiography. More research is warranted.
